# Canal of Nuck cyst in adulthood: A rare differential for inguinal masses – A case report

**DOI:** 10.1016/j.ijscr.2025.111568

**Published:** 2025-06-26

**Authors:** Jasser Rchidi, Yassine Kallel, Ghazi Laamiri, Hazem Alouani, Mahdi Bouassida, Hassen Touinsi

**Affiliations:** Department of General Surgery, Hospital Mohamed Taher Maamouri, Nabeul, Tunisia

**Keywords:** Canal of Nuck, Inguinal mass, Female hydrocele, Hernia

## Abstract

**Introduction:**

Canal of Nuck defects are rare anomalies of the female genitalia, typically diagnosed in childhood, but may present in adulthood as inguinal masses. These defects result from the incomplete obliteration of the canal of Nuck, which is analogous to the male processus vaginalis, leading to cyst formation or herniation of intra-abdominal contents. The condition is often misdiagnosed due to its nonspecific presentation.

**Case presentation:**

We present a case of a patient with a Nuck canal cyst, initially misdiagnosed as a lipoma. She presented with right groin pain and a palpable mass, which was confirmed to be a canal of Nuck cyst via ultrasound. Surgical excision was performed, followed by histopathological confirmation.

**Discussion:**

This case highlights the importance of considering canal of Nuck defects in the differential diagnosis of inguinal masses in adult females, given the potential for misdiagnosis and delayed treatment. Imaging plays a key role in diagnosis, aiding in distinguishing this condition from other inguinal masses. Early surgical intervention offers favorable outcomes and prevents complications.

**Conclusion:**

Canal of Nuck cysts, although rare, should be included in the differential diagnosis of inguinal masses in adult females. Accurate imaging and timely surgical excision are essential for effective management and positive patient outcomes.

## Highlights

• Canal of Nuck cysts are rare and often misdiagnosed inguinal masses in adult females.
• Canal of Nuck cysts often presents with groin pain and a palpable mass
• Ultrasound and MRI are key imaging modalities for accurate diagnosis.
• Definitive treatment is surgical excision with histopathological confirmation.
• Misdiagnosis can delay treatment and increase morbidity.

## 1 Introduction

Defects in the canal of Nuck are rare abnormalities of the female genitalia, typically diagnosed in young girls, often within the first five years of life [1]. Anton Nuck described the first recorded case in 1691. Though uncommon, this condition is frequently underdiagnosed and predominantly affects females. It results from the incomplete obliteration of the canal of Nuck, an embryonic structure analogous to the male processus vaginalis. This failure to close can lead to the formation of a cyst or the herniation of intra-abdominal structures through the patent canal. Symptoms are often nonspecific and may resemble other pathologies, such as inguinal or femoral hernias. A Nuck canal cyst typically presents as a benign swelling, which may be either painless or painful, in the inguinal region, sometimes extending to the labia majora. The incidence rate is approximately 0.76 % [2]. Misdiagnosis is common, with the condition often being mistaken for a lipoma, lymph node, or hernia, delaying or leading to inappropriate treatment. This case report presents a 25-year-old woman diagnosed with a Nuck canal cyst, highlighting the diagnostic methods, surgical intervention, and histological findings.

## 2 Case presentation

A 25-year-old female presented with persistent right groin pain lasting several months. She initially noticed a small lump in the right inguinal region, which was diagnosed as a lipoma based on clinical examination. At the time, the mass was asymptomatic and did not interfere with her daily activities. There was no history of similar swelling during childhood or adolescence. Over several months, the patient experienced progressive enlargement of the lesion with increasing discomfort. Eventually, the pain became severe enough to limit her daily activities, prompting her to seek medical attention.

**Fig. 1:**
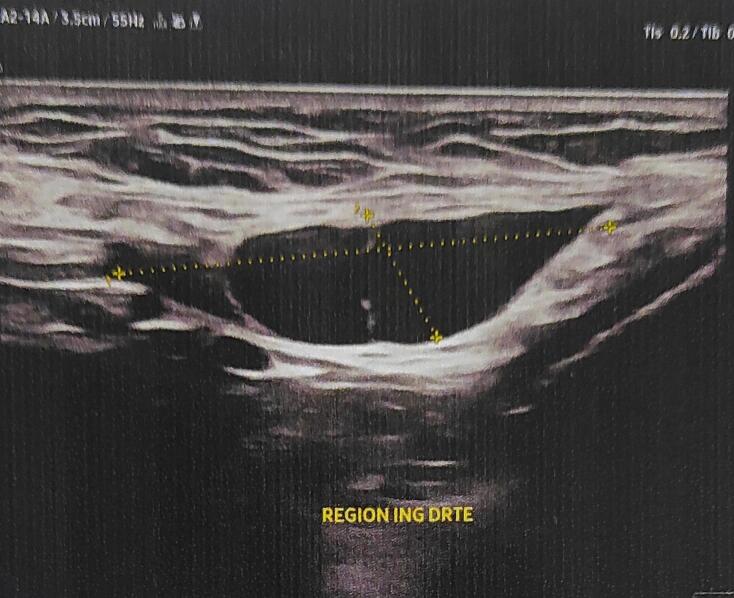
Ultrasound (US) that revealed a cystic structure of 4.1 × 2.3 cm in size with internal ipoecogenic mass.

Despite multiple consultations, no definitive diagnosis was made, and conservative management was recommended. However, due to the worsening pain and persistent swelling, further evaluation was sought. Clinical examination revealed a firm, non-tender, 4 cm mass in the right groin, mobile and non-adherent to the overlying skin, with no signs of inflammation. An ultrasound revealed a well-defined, cystic lesion measuring 40 mm in diameter, extending from the inguinal canal toward the labia majora Fig. 1. These findings suggested a canal of Nuck cyst, and the patient was scheduled for elective excision.

**Fig. 2:**
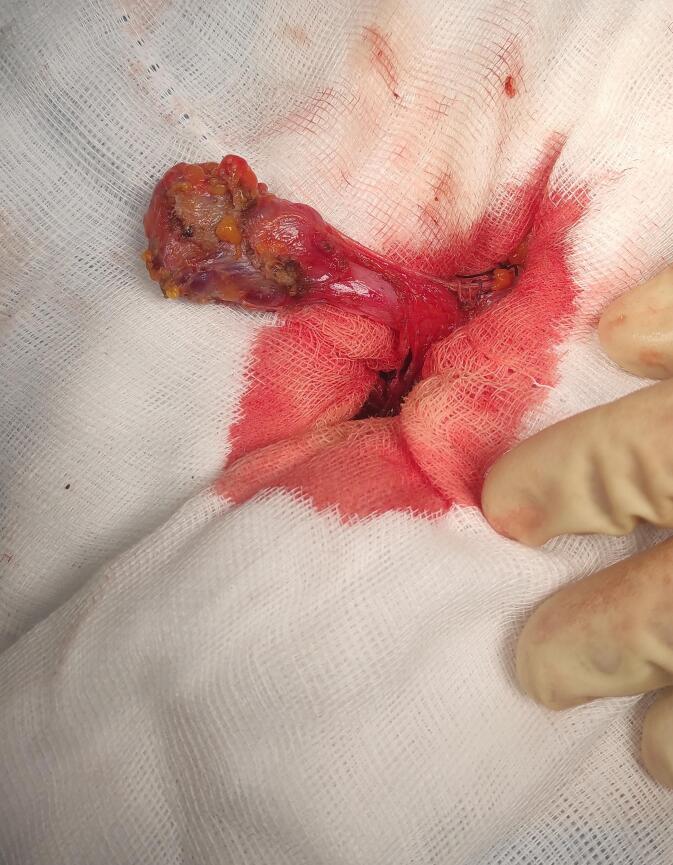
Intraoperative image of the cyst of the canal of Nuck.

**Fig. 3:**
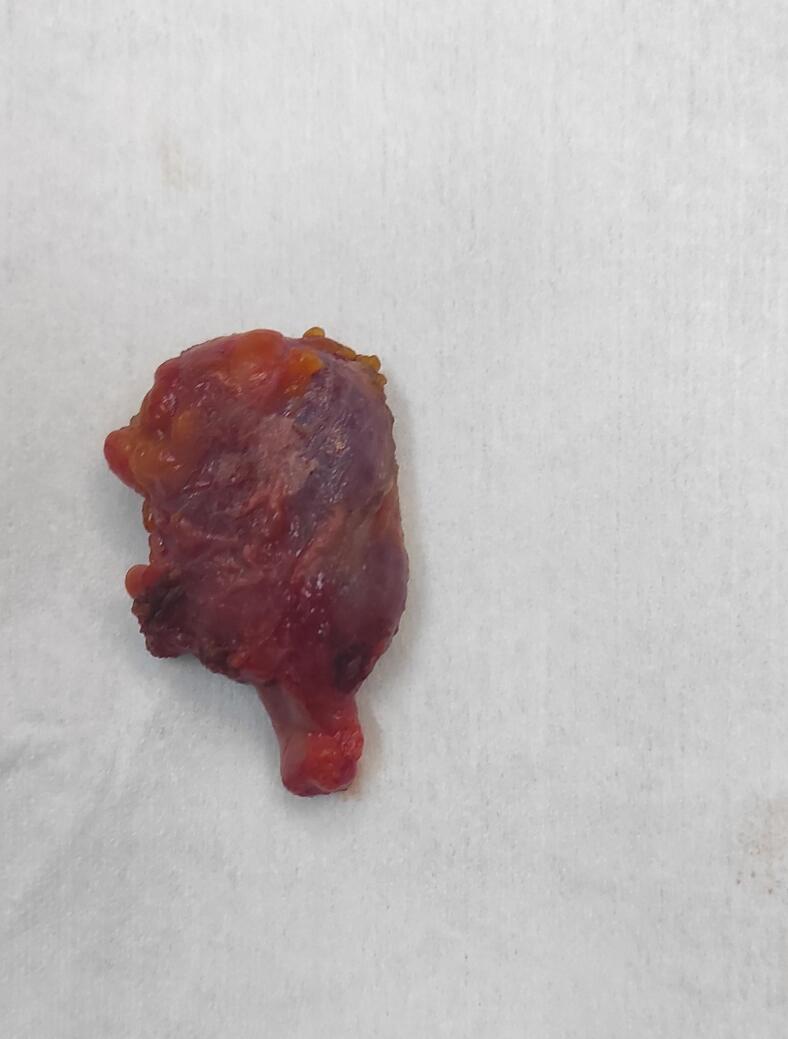
Picture after the dissection of the cyst from the round ligament.

During surgery, a 4 cm cystic structure extending from the peritoneal cavity through the inguinal canal was identified. The lesion was carefully dissected from the round ligament, and the proximal portion of the canal was ligated at the deep inguinal ring. No associated hernias were found. The surgical site was closed in layers, and the patient recovered uneventfully Fig. 2, Fig. 3. Histopathological analysis confirmed a cyst lined with mesothelium and surrounded by connective tissue, consistent with a cyst of canal of Nuck. At six and twelve months follow-ups, the patient was asymptomatic, with no recurrence.

This work has been reported in compliance with the SCARE 2020 criteria for surgical case reports [3].

## 3 Discussion

The canal of Nuck hydrocele remains a rare and often misdiagnosed condition, frequently mistaken for inguinal or femoral hernias [4]. While cases in adult females are exceedingly rare, misdiagnosis can delay treatment, leading to complications. To date, limited literature exists on adult Nuck hydrocele, with no comprehensive case series specifically focusing on adult females. This case aims to address this gap, providing valuable insights for clinicians involved in diagnosing and managing this uncommon condition [5].

Several differential diagnoses should be considered when evaluating inguinal masses in females, including hernias, lymphadenopathy, abscesses, Bartholin's cysts, hematomas, and endometriosis [6,7]. Among these, hydroceles and hernias are most frequently identified in the canal of Nuck [8]. Given its underrecognized prevalence, Nuck's hydrocele should be included in the differential diagnosis when assessing patients with groin pain or unusual swelling. Early recognition of this rare condition can significantly improve clinical outcomes.

Imaging plays a critical role in diagnosing Nuck's hydrocele [9]. Ultrasound is a cost-effective, reliable modality for differentiating this condition from others with similar symptoms. On ultrasound, the Nuck cyst appears as a thin-walled, well-defined, tubular or dumbbell-shaped structure, typically anechoic or hypoechoic. Color Doppler imaging reveals no internal vascularity, which helps distinguish it from other lesions [10]. If the diagnosis remains unclear or a hernia is suspected, magnetic resonance imaging (MRI) provides further valuable information. On MRI, a hydrocele in the canal of Nuck appears as a cystic structure within the inguinal canal, hypointense on T1-weighted images and hyperintense on T2-weighted images. Fine septations with mild enhancement may suggest underlying inflammation or infection [11]. The Valsalva maneuver can also help differentiate between a hernia and a hydrocele, as hernias show dynamic changes, while a hydrocele remains stable [12].

A definitive diagnosis requires surgical intervention followed by histological analysis. Although many cases of Nuck's cyst are associated with an inguinal defect, this is not always the case, as demonstrated by our patient. The cyst results from the persistence of the processus vaginalis, which typically closes after birth. When the canal remains patent, cyst formation can occur without a significant hernial defect. Surgical management includes cyst resection and, if necessary, hernia repair [13]. Both open and laparoscopic excision techniques are used, with mesh reinforcement for hernia repair. Transabdominal preperitoneal (TAPP) and Lichtenstein hernioplasty are both effective, with TAPP possibly reducing postoperative pain [14]. The laparoscopic total extraperitoneal (TEP) approach is a viable alternative, though more technically challenging [15]. For patients not candidates for surgery, ultrasound-guided cyst aspiration offers temporary relief [16].

## 4 Conclusion

The hydrocele of the canal of Nuck is a rare and often misdiagnosed condition, typically confused with inguinal hernias or abscesses. Its underrecognition emphasizes the need for increased awareness among clinicians. A comprehensive understanding of the anatomy, pathology, and clinical manifestations of this condition is crucial for improving diagnostic approaches and ensuring early detection. Ultrasonography is a useful, cost-effective modality for distinguishing Nuck's hydrocele from other conditions. When an inguinal hernia is present, the treatment of choice involves hydrocelectomy followed by hernioplasty. Early recognition and accurate diagnosis can optimize management, reduce patient morbidity, and enhance surgical outcomes.

## Authors' contributions

**Conceptualization:** Jasser Rchidi, Yassine Kallel.

**Data collection:** Hazem Alouani, Ghazi Laamiri.

**Supervision:** Hassen Touinsi.

**Data analysis:** Mahdi Bouassida.

**Writing-original draft:** Jasser Rchidi, Hazem Alouani.

**Writing-review and editing:** Yassine Kallel, Ghazi Laamiri.

## Patient consent

Written informed consent was obtained from the patient for publication of this case report and accompanying images.

## Ethical approval

Not required for single-patient case reports in our institution.

## Guarantor

Jasser Rchidi.

## Patient perspective

The patient expressed satisfaction with the surgical outcome and experienced a good recovery.

## Funding

This research did not receive any specific grant from funding agencies in the public, commercial, or not-for-profit sectors.

## Declaration of competing interest

The authors declare that there is no conflicts of interest.
